# Long-Term Outcomes and a Radiological Assessment of Hydroxyapatite–Tricalcium Phosphate-Coated Total Hip Arthroplasty (Trilogy/Zimmer): A Long-Term Follow-Up Study

**DOI:** 10.3390/medicina60071154

**Published:** 2024-07-17

**Authors:** Shinya Ibuchi, Norio Imai, Yoji Horigome, Yuki Hirano, Keishi Kimura, Hiroyuki Kawashima

**Affiliations:** 1 Division of Orthopedic Surgery, Department of Regenerative and Transplant Medicine, Niigata University Graduate School of Medical and Dental Sciences, Niigata 951-8510, Japan; ibuchi920shinya@msn.com (S.I.);; 2 Department of Orthopedic Surgery, Uonuma Kikan Hospital, Niigata 949-7302, Japan; 3 Division of Comprehensive Musculoskeletal Medicine, Niigata University Graduate School of Medical and Dental Sciences, Niigata 951-8510, Japan

**Keywords:** total hip arthroplasty, hydroxyapatites, tricalcium phosphate, survival rate, follow-up studies

## Abstract

*Background and Objectives:* Favorable short- and mid-term results for hydroxyapatite (HA)–tricalcium phosphate (TCP)-coated total hip arthroplasty (THA) (Trilogy/Zimmer) have been reported in the literature; however, the long-term results beyond 15 years have not been documented. Therefore, this study evaluated the long-term postoperative results, radiological bone changes, and implant fixation of the acetabular component of HA-TCP-coated THA. *Materials and Methods:* This is a retrospective cohort study of 212 patients who underwent primary HA-TCP-coated THA (Trilogy/Zimmer) at our institution between 1 October 2002, and 31 March 2008; 166 who were available for follow-up at least 15 years postoperatively were included (capture rate: 78.3%). All implants were Trilogy/Zimmer. We investigated the survival rate, with aseptic loosening as the endpoint. Clinical evaluations included the presence of dislocation and a modified Harris Hip Score (mHHS) preoperatively and at the final observation. *Results:* The mean age at surgery and at the follow-up period were 57.7 ± 9.6 and 17.1 ± 1.5 years, respectively. The survival rate was 99.4% (165/166), with aseptic loosening as the endpoint. Dislocation was observed in 4/166 (2.4%) patients. The mHHS improved significantly from 46.1 points preoperatively to 82.2 points during the last survey (*p* < 0.05). The results revealed that fixation was favorable in all cases except for one case of aseptic loosening. The Trilogy implant coated with HA-TCP was highly effective in bone induction, and bone ingrowth was considered to have occurred without failure, further indicating its usefulness. The long-term results of cementless THA using an HA-TCP coating (Trilogy/Zimmer), with a mean follow-up period of 17.1 years, revealed a commendable survival rate of 99.4%, considering aseptic loosening as the endpoint. *Conclusions:* HA-TCP-coated THA (Trilogy/Zimmer) had good long-term results. However, further long-term observation is required in patients who have undergone this surgery, and the stem side should be evaluated and investigated, including comorbidities.

## 1. Introduction

Recently, reports on the clinical results of cementless total hip arthroplasty (THA) have demonstrated that its 10-year survival rate is comparable to that of cemented THA, which has a long history of favorable long-term results [1].

Kawaji et al. [2] used an implant (Trilogy/Zimmer), which was designed by coating the stem and cup with hydroxyapatite (HA) and tricalcium phosphate (TCP). They reported that over a mean follow-up period of 6.9 years, only one of the 108 joints had a loosened acetabular component. However, long-term results (>15 years) have not been documented.

We hypothesized that HA-TCP-coated THA (Trilogy/Zimmer) would have good long-term results. To investigate this, we evaluated the long-term postoperative results, radiological bone changes, and implant fixation success of the acetabular component of HA-TCP-coated THA (Trilogy/Zimmer). We report the long-term postoperative results, radiological evaluation of bone changes, and implant fixation in HA-TCP-coated THA (Trilogy/Zimmer).

## 2. Materials and Methods

### 2.1. Ethics Statements

The Institutional Review Board of Niigata University approved the study design (approval number: 2023-0283) and waived the requirement for informed consent because of the study’s cross-sectional, retrospective, and non-interventional nature. All methods used in this study were performed according to the relevant guidelines and regulations.

### 2.2. Study Design and Population

Of the 212 patients who underwent primary HA-TCP-coated THA (Trilogy/Zimmer) at our institution between 1 October 2002, and 31 March 2008, 166 who were available for follow-up for at least 15 years postoperatively were included in this study (capture rate: 78.3%). There were no excluded cases in this study.

We set the survival rate with aseptic loosening as the endpoint, and the absence of cup loosening at the last observation was considered implant fixation success. Clinical evaluation was performed using the modified Harris Hip Score (mHHS) [3] in the patient’s medical records, preoperatively and at the last follow-up. Additionally, patient history reporting any dislocation event was investigated.

A multi-slice computed tomography (CT) scanner with a 64-row detector (Aquilion 64TM, Toshiba Medical Systems, Otawara, Tochigi, Japan) was used to acquire approximately 600 slices (slice thickness: 1.25 mm) from each limb.

### 2.3. Surgical Procedure

Four experienced surgeons performed all surgeries using the direct lateral, anterolateral supine, or Orthopädische Chirurgie of München approach with patients under general anesthesia. The surgeon determined the surgical approach based on the time of the year. Using a CT-based navigation system (VectorVision Hip^®^, Brainlab AG Munich, Germany) and highly crosslinked polyethylene (Longevity^®^, Zimmer Biomet, Warsaw, IN, USA), the uncemented acetabular component Trilogy^®^ (Zimmer Biomet, Warsaw, IN, USA) was inserted. We selected the femoral component from three types of implants, namely, the VerSys Midcoat collarless stem^®^ in 92 hips, the Fiber metal taper^®^ in 65 hips, and the Cemented^®^ (Zimmer Biomet, Warsaw, IN, USA) in 9 hips, adjusted to the configuration of each femur. The diameters of the femoral heads, made of zirconium, were 22, 26, and 28 mm in 7, 106, and 53 hips, respectively.

### 2.4. Data Collection

We used ZedView^®^ software, Version 16.0.0.0 (Lexi, Tokyo, Japan) to measure the implantation angle, namely, the inclination and anteversion of the acetabular component, after creating a three-dimensional (3D) digital bone model from CT taken 1 week postoperatively [4].

We reconstructed 3D pelvic and femur bone surface models from the CT data of each patient. To measure the orientation of the acetabular component, such as the inclination and anteversion, we adjusted the pelvis model to the anterior pelvic plane (APP). The bilateral anterior iliac spines and pubic symphysis were on the same horizontal plane [5]. Finally, the 3D model of the femur was adjusted to the retrocondylar plane (RCP), as previously reported [6].

### 2.5. Clinical Evaluation

The patients were followed up after 1 month, 3 months, 6 months, and 1 year, and every year thereafter postoperatively. We examined the anteroposterior plain radiograph of the bilateral hips, and the mHHS was obtained at each follow-up. The mHHS was evaluated preoperatively and during the last visit to our institution. We also evaluated cup loosening based on the demarcation grade of the acetabular component using plain radiographs, according to the Hodgkinson classification method [7]. The cups were considered loose in the Hodgkinson classification types 3 and 4.

### 2.6. Statistical Analysis

The data were analyzed using IBM SPSS Statistics for Windows, version 21 (IBM Corp., Armonk, N.Y., USA). We compared the pre- and final mHHS scores using Student’s *t*-test. The validity of the measured values was also analyzed. Intraobserver and interobserver reliability with intraclass correlation coefficients and two-sided 95% confidence intervals were calculated to evaluate the validations. We measured the values two times at >1-week intervals to determine intraobserver reliability. Furthermore, we compared our measurements with those conducted by another observer to assess interobserver reliability. Statistical significance was set at *p* < 0.01.

## 3. Results

All implants were Zimmer Trilogy acetabular multihole cups, and the surgical approaches used were the lateral, anterolateral supine, and Orthopädische Chirurgie of München approaches in 109, 32, and 25 cases, respectively. Intraoperative support was provided in 146 and 20 patients with and without CT-based navigation (VectorVision Hip^®^, Brainlab AG, Munich, Germany), respectively (Table 1).

The mean age at surgery and at the follow-up period were 57.7 ± 9.6 and 17.1 ± 1.5 years, respectively. The underlying diseases were osteoarthritis of the hip joint, necrosis of the femoral head, rheumatoid arthritis, and pigmented villonodular synovitis in 136, 23, 6, and 1 patients, respectively (Table 1).

We identified one case of revision because of aseptic loosening, and the survival rate with aseptic loosening as the endpoint was 99.4% (165/166). Dislocation occurred in 4/166 (2.4%) patients, with three cases within 1 month postoperatively and one after 7 years. The cases of delayed dislocation were repetitive dislocations. The mHHS improved significantly from 46.1 points preoperatively to 82.2 points during the last survey (*p* < 0.05).

**Figure 1:**
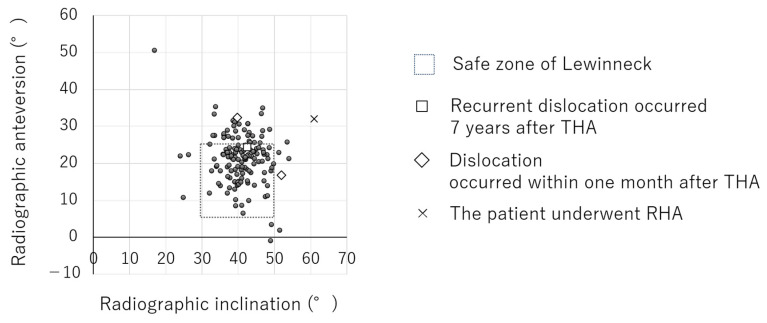
Radiographic inclination and anteversion measured using ZedView Version 16.0.0.0. In total, 127 cases (76.5%) were within the Lewinnek safe zone [5].

The radiographic inclination and anteversion measured using the ZedHip were 41.1° and 21.1° relative to the APP, respectively. By contrast, the stem anteversion was 19.1° relative to RCP (Table 2). Overall, 127 (76.5%) cases were within the Lewinnek safe zone [5] (Figure 1).

The reliability of the measured angles was high, with both intra- and inter-rater reliability exceeding 0.9 (Table 3).

The evaluation of fixation in 165 cases, excluding the aseptic loosening cases, was as follows: Hodgkinson classification type 0, 161 cases; type 1, four cases; type 2, zero cases; and type 3, zero cases (Table 2).

*Case Presentation*

**Figure 2:**
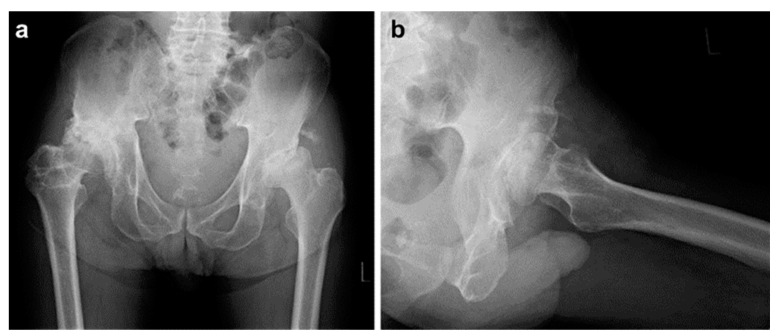
Preoperatively: A 62-year-old male patient was diagnosed with necrosis of the left femoral head. (**a**) Preoperative anteroposterior plain radiograph of the bilateral hips. (**b**) Preoperative axial image radiograph of the left hip.

**Figure 3:**
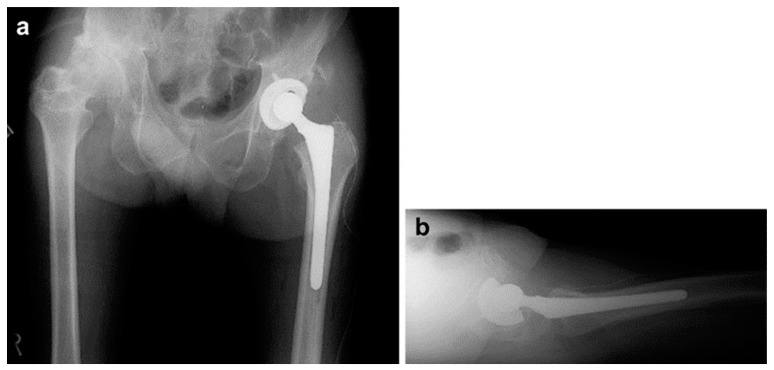
Primary total hip arthroplasty was performed. (**a**) An anteroposterior plain radiograph of the bilateral hips immediately after primary surgery. (**b**) Axial image radiograph of the left hip immediately after primary surgery.

**Figure 4:**
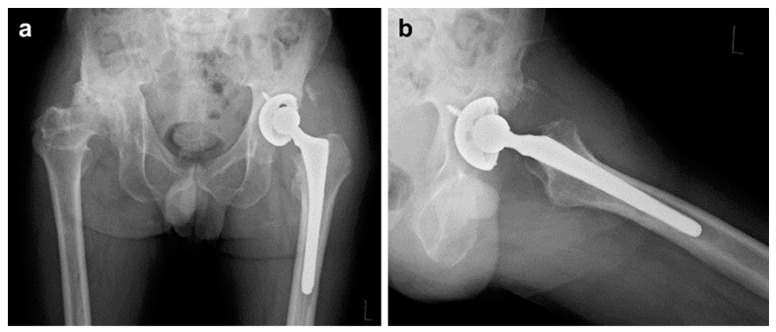
Four months after the primary surgery, cup loosening and displacement were observed. (**a**) An anteroposterior plain radiograph of the bilateral hips at 4 months after primary surgery. (**b**) Axial image radiograph of the left hip at 4 months after primary surgery.

One case of revision THA included a 62-year-old male patient who underwent THA via a lateral approach for necrosis of the left femoral head (Figure 2a,b). Four months after the primary surgery (Figure 3a,b), cup loosening and displacement were observed (Figure 4a,b). The blood test results and physical findings indicated no infection during the disease course, and revision surgery was performed.

## 4. Discussion

In this study, we evaluated the long-term postoperative results, radiological bone changes, and implant fixation success of the acetabular component of HA-TCP-coated THA. We included 166 patients with a mean follow-up of 17.1 years, resulting in a survival rate of 99.4%, with aseptic loosening as the endpoint. Clinically, the mHHS improved significantly (*p* < 0.05) from 46.1 points preoperatively to 82.2 points during the last survey, with 161 patients having Hodgkinson classification type 0, four having type 1, and zero having type 2 or 3 on the frontal X-ray hip image. Except for one revision because of aseptic loosening, implant fixation was considered successful because of the absence of cup loosening at the last observation, underscoring the effectiveness of the HA-TCP-coated trilogy for bone induction. Bone ingrowth was considered to have occurred without failure, indicating the usefulness of HA-TCP.

In the epidemiological data of THA by Ishan et al. [8], the mean age of patients undergoing primary THA (2,838,742 patients) was 65.98 years. However, the mean age during surgery in this study was 57.7 ± 9.6 years, which is less than that of the epidemiology report for THA mentioned above. Thus, many patients were active and had returned to their usual routines, including work, with little or no cup loosening, which is excellent.

Currently, whether cemented THA or cementless THA is superior remains controversial. In this study, we collected hip arthroplasty data from the National Joint Registry 18th Annual Report published in 2021 and documented primary hip operations performed between 1 April 2003, and 31 December 2020. Aseptic loosening was the most common reason for revision in all patients. In evaluating the number of revisions per 1000 prosthesis years regarding aseptic loosening, the rate was 1.5 and 1.31 for all-cemented and uncemented, respectively [9]. Macheras et al. reported that implanted all-poly cemented cups had a higher risk of aseptic loosening than press-fit porous-coated uncemented cups from 1980 to 2001. However, no significant difference was observed in the risk between these two design concepts when the endpoint was defined as any revision (including the exchange of liner) [10]. Marco et al. demonstrated better surgical and clinical outcomes in hip replacement surgery for femoral neck fracture in both cementless and cemented stem groups, with satisfactory results in both groups of the total study cohort. At the mid-term follow-up, patients with cementless stem implants had a higher complication rate than that of patients with cemented prostheses [11]. Okutani et al. reported that the 20-year survival rate with cemented socket revision for aseptic loosening was 92.4% (95% confidence interval: 87.6–95.5%) according to the Kaplan–Meier survival analysis [12]. However, the use of cement has the risk of complications of bone cement implantation syndrome (BCIS), an immediate complication of cementing components that occurs during THA when medullary fat is forced into the blood vessels by intramedullary pressure from cementation [8]. Cardiopulmonary disease and bony metastases create high permeability and vascularity on the bone surface, increasing the risk of BCIS [13]. Sequelae of BCIS include systemic hypotension, hypoxemia, pulmonary hypertension, arrhythmias, and cardiac arrest. Intraoperative mortality from BCIS is rare, as evidenced by a previous study that reported 23 deaths among the 38,488 THAs performed at one institution over 33 years [14].

Good short- and mid-term results for HA-TCP-coated THA have been reported in cementless THA [2,15,16]. Kawaji et al. investigated 108 (11 males and 97 females; mean age: 64.6 years) hip joints over a mean follow-up period of 6.9 years and reported that the underlying diseases included secondary osteoarthritis in 87 joints, idiopathic osteonecrosis of the femoral head in 16 joints, rapidly destructive coxarthrosis in 4 joints, and idiopathic ossification of the labrum in 1 joint. At the final follow-up, loosening of the acetabular component was observed in one case but with no reported dislocation. Pain scores using the Japan Orthopedic Association scoring system and range of motion tests showed statistically significant improvement following THA [2].

Shahcheraghi et al. reported that all Versys-Trilogy prostheses survived in a mean follow-up of 49 (range: 26–78) months, with no radiographical or clinical evidence of loosening or wear [15]. Shetty et al. reported the survival of 134 consecutive HA-coated uncemented THA over a mean follow-up of 14.2 (range: 13–15) years. The DeLee and Charnley zones for the cup were evaluated in radiographs, and no loosening of the cup was observed during the study period, suggesting that the long-term results of these HA-coated prostheses were satisfactory [16].

Surdam et al. reported that in 258 cases of Zimmer’s porous-coated cup without HA-TCP coating after a mean follow-up duration of 9 years, 9 cases of cup-side complications required revision, among which osteolysis was observed in 13% [17]. Thanner et al. performed a matched pair test of 23 HA-TCP-coated and uncoated cups in Zimmer’s cups and showed that the HA-TCP-coated cups did not differ from the uncoated cups in terms of clinical results. However, radiographically, both the cup inclination progression and radiolucent line decreased over time, and the superiority of HA-TCP-coated cups was reported 2 years after the operation [18]. Thanner et al. also compared the same HA-TCP-coated cups with and without screw fixation and reported that the fixation of HA-TCP-coated cups was sufficient even without a screw [18]. However, only a few reports have been published on long-term results beyond 15 years. Therefore, this study evaluated the long-term results of HA-TCP-coated THA (Trilogy/Zimmer) in the acetabular component for over 15 years.

Recently, 3D porous cups with a high coefficient of friction and porosity have been developed by many companies for use in clinical practice to improve initial fixation. These 3D porous cups are made of metal and have good bone affinity, a porous surface shape, a high coefficient of friction, and are expected to have strong initial fixation [19]. They are also believed to promote good bone ingrowth from an early stage [14]. Various companies have developed different products, and good results have been obtained in clinical trials for 3D porous cups [20,21,22]. Conversely, Carli et al. reported 109 hips that underwent primary THA using a 3D porous cup manufactured by Stryker and were followed up over a mean period of 4.2 years. In this study, two patients required revision surgery by loosening, and 30.3% exhibited radiolucent lines [23]. In a comparison of 3D porous-surfaced and HA-coated cups, Imai et al. compared the outcomes of 101 3D-printed (SQRUM TT) and 35 HA-coated (SQRUM HA) cups with the same stem and bearing in patients with mild dysplasia. The short-term clinical outcomes significantly improved in both cohorts, achieving similar results. However, the HA-coated cups had a lower rate of radiolucent lines than the 3D-printed cups in the 2-year radiographical outcomes [24].

The results of our study are comparable to those of other studies and are considered good. Moreover, 4/166 (2.4%) patients exhibited demarcation on the outer side of the cup, suggesting that HA-TCP forms a strong bond with the bone. However, the stress shielding in the unloaded area was rather advanced. One revision surgery was performed 4 months after the primary surgery because of loosening. Because the blood test results and physical findings indicated no infection during the procedure, poor initial fixation was considered the cause; however, poor placement angles may have also contributed. Therefore, long-term observation of patients is required following this type of surgery.

### Limitations

Regarding this study’s limitations, the number of patients included in this study was small (n = 166), and this study was not compared with other implant studies, nor was the stem side evaluated. Many of the cases that could not be followed up (46/212 cases) involved transfers due to patient relocation, dropouts, death, or cases with unknown details. Additionally, clinical scores were evaluated only for the mHHS. Moreover, the mHHS at the final evaluation was 82.2, which is not high compared to that reported in other studies [15,16]. The mHHS was not high, which may have been influenced by the extended 20-year follow-up and the patients’ advanced age (>80 years) at the last observation. Therefore, future investigations should consider that medical conditions, including heart failure, chronic obstructive pulmonary disease, knee osteoarthritis, and frailty, may impact the mHHS. Furthermore, the stem side should be evaluated and investigated, including comorbidities.

## 5. Conclusions

The long-term results of cementless THA using HA-TCP-coated THA (Trilogy/Zimmer) in our department revealed that the mean follow-up was 17.1 years, showing a commendable survival rate of 99.4%, considering aseptic loosening as the endpoint. In cementless THA, the long-term results of HA-TCP-coated THA were generally good. However, further long-term observation is required for patients who have undergone this surgery.

## Figures and Tables

**Table 1:** Case summary.

Cases	166/212 cases (capture rate: 78.3%). 15 males and 151 females
Mean age at surgery	57.7 ± 9.6 years
Observation period	17.1 ± 1.5 years
Underlying diseases	Osteoarthritis of the hip 136 cases Necrosis of the femoral head 23 cases Rheumatoid arthritis 6 cases Pigmented villonodular synovitis (PVS) 1 case
Surgical approach	Lateral approach (DL) 109 cases Anterolateral supine approach (ALS) 32 cases Orthopädische Chirurgie of München approach (OCM) 25 cases
Intraoperative support	None 20 cases CT-based navigation (Brainlab VectorHip) 146 cases

CT: computed tomography.

**Table 2:** Results.

Survival rate	165/166 (99.4%)
Dislocation	4/166 (2.4%)
mHHS (preop/postop)	46.1 ± 12.2/82.2 ± 15.8 *
RA (°)	41.1 ± 5.8
RI (°)	21.3 ± 6.9
SA (°)	19.1 ± 14.1
Demarcation ** (Hodgkinson classification)	Type 0 161 Type 1 4 Type 2 0 Type 3 0

* *p* < 0.05; ** 165 cases excluding one revision surgery. mHHS: modified Harris Hip Score; preop: preoperatively; postop: postoperatively.

**Table 3:** The reliability of the measured angles.

**Intra-Rater Reliability**				
	MAD	ICC	95% CI	*p*-value
RA (°)	1.3 ± 0.8	0.975	0.967–0.982	<0.001
RI (°)	1.2 ± 0.9	0.976	0.968–0.983	<0.001
SA (°)	0.9 ± 1.5	0.963	0.941–0.985	<0.001
**Inter-Rater Reliability**				
	MAD	ICC	95%CI	*p*-value
RA (°)	1.8 ± 1.4	0.936	0.914–0.953	<0.001
RI (°)	2.2 ± 1.7	0.947	0.928–0.961	<0.001
SA (°)	2.0 ± 2.1	0.955	0.939–0.967	<0.001

MAD: mean absolute difference; ICC: inter-class correlation coefficient; CI: confidence interval.

## Data Availability

The original contributions presented in the study are included in the article.
